# A panel of miRNAs derived from plasma extracellular vesicles as novel diagnostic biomarkers of lung adenocarcinoma

**DOI:** 10.1002/2211-5463.12753

**Published:** 2019-11-21

**Authors:** Bing Yao, Shuang Qu, Ruifeng Hu, Wen Gao, Shidai Jin, Ming Liu, Quan Zhao

**Affiliations:** ^1^ The State Key Laboratory of Pharmaceutical Biotechnology School of Life Sciences Nanjing University China; ^2^ Department of Oncology The First Affiliated Hospital of Nanjing Medical University China

**Keywords:** biomarker, extracellular vesicles, lung adenocarcinoma, miRNAs

## Abstract

Lung cancer is the leading cause of cancer‐related morbidity and mortality worldwide, with lung adenocarcinoma (LUAD) being the most common histological subtype (approximately 40%). In the absence of reliable screening biomarkers for early diagnosis, most patients with LUAD are inevitably diagnosed at an advanced stage. MicroRNAs (miRNAs) encapsulated within plasma‐derived extracellular vesicles (EVs) may be suitable for use as noninvasive diagnostic biomarkers for aggressive malignancies, including LUAD. In this study, we first investigated the miRNA profiles of plasma‐derived EVs from LUAD patients and healthy donors, and then systematically evaluated the expression patterns of selected plasma‐derived EV miRNAs in a large cohort of patients with LUAD and healthy controls. Notably, we observed that miR‐451a, miR‐194‐5p, and miR‐486‐5p were significantly increased in EVs from LUAD patients, compared to healthy controls. The area under the curve values for the three miRNAs were 0.9040 (95% confidence interval [CI], 0.8633–0.9447) for miR‐451a, 0.7492 (95% CI, 0.6992–0.7992) for miR‐194‐5p, and 0.9574 (95% CI, 0.9378–0.9769) for miR‐486‐5p, while the AUC of the combination of these three miRNAs was 0.9650. Thus, these results suggest that these EV miRNAs may be promising candidates for the development of highly effective, noninvasive biomarkers for early LUAD diagnosis.

## Abstract 2

Lung cancer is the leading cause of cancer‐related morbidity and mortality worldwide, with lung adenocarcinoma (LUAD) being the most common histological subtype (approximately 40%). In this study, we observed that miR‐451a, miR‐194‐5p, and miR‐486‐5p in extracellular vesicles derived from plasma may be suitable as highly effective and noninvasive biomarkers for early LUAD diagnosis.

## Graphical abstract

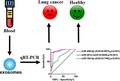

**Abbreviations**
AUC: the area under the curve values
dNTPs: deoxyribonucleotide triphosphates
EV: extracellular vesicle
LUAD: lung adenocarcinoma
miRNA: microRNA
NTA: Nanoparticle tracking analysis
ROC: Receiver operating characteristic curve
RT‐qPCR: Quantitative real‐time Polymerase Chain Reaction
TEM: Transmission electron microscopy

Lung cancer is the leading cause of cancer‐related death worldwide 1. Lung adenocarcinoma (LUAD) accounts for approximately 40% of all lung cancers and causes more than 500 000 deaths every year 1. Unfortunately, the incidence of LUAD is sharply increasing, especially in females and never‐smokers 2. Moreover, the 5‐year survival rate of LUAD is just 15% due to advanced‐stage diagnosis and delayed treatment. Efforts and techniques for early identification and intervention in LUAD have strengthened the clinical diagnosis through low‐dose CT scans, but have not been shown to be sufficiently effective in either early detecting lung cancer or significantly reducing LUAD mortality 3. European studies showed that low‐dose CT scans might increase cancer risk and cost by repeated exposure to ionizing radiation 4. The survival of patients with LUAD is fully proved to be closely associated with the tumor stage at initial diagnosis. Therefore, novel noninvasive and effective biomarkers for early detection of LUAD are urgently needed.

MicroRNAs (miRNAs) are 22‐nt, small noncoding RNAs that negatively regulate gene expression and play important roles in multiple physiological and pathological processes, especially cancers 5. More interestingly, several studies revealed that miRNAs are stably present in serum/plasma (named circulating miRNAs) and that their expression patterns are closely correlated with progression and prognosis in a variety of tumors, including lung cancer 6, 7. Circulating miRNAs are considered to be novel promising diagnostic and prognostic biomarkers for cancer screening 8. Nevertheless, circulating miRNAs are easily affected by miRNAs released by circulating dysfunctional cells, such as platelets and red blood cells 8. Extracellular vesicles (EVs) in the plasma/serum contain lots of nucleotides, including miRNAs, and proteins 8, 9. Compared to circulating miRNAs, miRNAs in EVs in serum/plasma are enriched and resistant to RNase‐mediated degradation 8, 9, 10, and have emerged as candidate biomarkers for many diseases. Consequently, the identification of EV miRNAs in plasma/serum shows great promise in developing biomarker‐based tools for cancer diagnosis.

To explore the new plasma EV miRNA signatures for LUAD, the miRNA profiles of EVs from LUAD patients and healthy donors were characterized to identify the different EV miRNAs. Then, these different EV miRNAs were verified in a large cohort of patients with LUAD and healthy controls by a quantitative real‐time polymerase chain reaction (RT‐qPCR) assay. The results showed miR‐451a, miR‐194‐5p, and miR‐486‐5p could be promising and noninvasive candidate biomarkers for diagnosing LUAD.

## Materials and methods

### Patient characteristics

Plasma samples from 434 patients newly diagnosed with LUAD without any treatment and 149 age, sex, and ethnicity‐matched healthy volunteers were recruited in this study according to protocols approved by the ethics committee of The First Affiliated Hospital of Nanjing Medical University. Paired preoperative and postoperative blood samples (*n* = 23) were collected from the stage I LUAD patients before surgery and at 1 month after resection. To avoid the influence of hemolysis and activated blood cells/platelets on the EV miRNAs, the samples from LUAD patients and healthy volunteers were strictly transported under 4° C, and the plasmas were isolated immediately once the blood was collected from the patients or healthy volunteers. To avoid the influence of hemolysis, the color of each sample was observed. The sample was discarded once hemolysis had occurred. The demographic and clinical features of the patients and healthy volunteers are summarized in Table 1. This study was approved by the Ethics Committee of Nanjing University, and written informed consent was obtained from all patients. All experiments were performed in accordance with relevant guidelines and regulations. Protocols were designed and performed according to the principles of the Helsinki Declaration.

**Table 1:** Demographic and clinical features of LUAD patients and healthy subjects.

Variable	Training set		Validation set		Blinded study		Before/after surgery
	LUAD	Normal	LUAD	Normal	LUAD	Normal	LUAD
	(*n* = 45)	(*n* = 45)	(*n* = 389)	(*n* = 104)	(*n* = 39)	(*n* = 39)	(*n* = 23)
	No.	No.	No.	No.	No.	No.	No.
Average age (years)	64.6 ± 9.8	65.2 ± 10.0	65.5 ± 9.9	69.4 ± 8.3	61.8 ± 7.1	62.4 ± 8.5	64.3 ± 6.7
Sex							
Female	21	25	205	59	21	20	13
Male	24	20	184	45	18	19	10
Stage							
I	23		216		18		23
II	11		94		10		
III	9		59		9		
IV	2		20		2		

### Plasma EV isolation and EV miRNA extraction

The Total Exosome Isolation Kit (from plasma) (Cat#4484450; Invitrogen, Carlsbad, CA, USA) was used to isolate the exosomes from plasma following the manufacturer’s protocol 11, 12. Briefly, blood samples collected into heparinized tubes were centrifuged at 3000 ***g*** for 10 min, and plasma specimens were stored in 1‐mL aliquots at –80 °C. Then, the EVs were isolated by the Total Exosome Isolation Kit. Briefly, plasmas were centrifuged at 1000 ***g*** for 20 min, 3000 ***g*** for 20 min, and 10 000 ***g*** for 20 min. Then, 1 mL of clarified plasma was transferred to a new tube and 0.5 volumes of 1× PBS was added. After mixing the sample thoroughly by vortexing, 0.2 volumes (i.e., Total volume = plasma + PBS) of the exosome precipitation reagent (from plasma) was added. Then, the mixture was incubated at room temperature for 10 min and followed by centrifugation at 10 000 ***g*** for 5 min. After the supernatant was discarded by pipetting, the pellet (EVs) was resuspended in 200 µL of 1× PBS for downstream analysis. For the extraction of the total RNAs in the EVs, the mirVana PARIS Kit (Ambion; Thermo Scientific, Shanghai, China) was used according to the manufacturer’s protocol. The synthetic *Caenorhabditis elegans* miRNA cel‐miR‐39 (5′‐UCACCGGGUGUAAAUCAGCUUG‐3µ) (RiboBio, Guangzhou, China) was spiked into the denatured exosomes as a normalization control 13.

### Nanoparticle tracking analysis and western blotting

Extracellular vesicles isolated from plasma were processed for nanoparticle tracking analysis (NTA) with a zetaview PMX 110 (Particle Metrix, Meerbusch, Germany) and its corresponding software (zetaview 8.02.28) according to the guidelines of the International Society for EVs 14, 15. Briefly, the instrument measured each sample at 11 different positions throughout the cell, and each position was read with two cycles. The mean, median, diameter sizes, and the concentration of the sample were calculated by the corresponding software. For each measurement, the instrument preacquisition parameters were set to a temperature of 23 °C, a sensitivity of 85, a frame rate of 30 frames per second, a shutter speed of 100, and a laser pulse duration equal to that of shutter duration. Postacquisition parameters were set to a minimum brightness of 25, a maximum size of 200 pixels, and a minimum size of 5 pixels. Polystyrene particles (MFCD00243243) from Merck (Darmstadt, Germany) with a known average size of 100 nm were used to calibrate the instrument before taking the sample readings. To characterize the EV protein marker CD63, EV protein was extracted with radioimmunoprecipitation assay buffer and western blot analysis was performed as previously described 10. CD63 was detected using an anti‐CD63 rabbit polyclonal antibody (1 : 1000; Abcam, Cambridge, UK). The bound proteins were visualized using ECL western blotting substrate (Thermo Fisher Scientific, Waltham, MA, USA), and band densities were analyzed with imagej software (National Institutes of Health, Baltimore, MD, USA).

### Transmission electron microscopy (TEM)

Transmission electron microscopy for EVs obtained from plasma samples was performed as previously reported 16. The EVs were resuspended in 1× PBS and applied to a carbon‐coated 200‐mesh copper grid for 20 min. Excess liquid at the edge was wicked off using filter paper. Subsequently, 2% phosphotungstic acid solution (HT152‐250ML; Sigma, San Francisco, CA, USA) was added to yield negative staining for 10 min at room temperature, and the copper grids were dried with the incandescent lamp. The microphotographs were obtained using a JEM‐1011 scanning transmission electron microscope (Hitachi, Tokyo, Japan).

### Illumina Hiseq 2500 analysis

Illumina Hiseq 2500 for EV miRNAs obtained from plasma samples was performed as previously reported 17, 18. One microgram of each RNA sample (five healthy controls and five LUAD) was used for miRNA library construction by the TruSeq Small RNA Library Prep kit (Illumina, San Diego, CA, USA) according to the manufacturer's instructions. Then, quantitative PCR (qPCR) was conducted using KAPA Library Quantification kit (KAPA Biosystems, Foster City, CA, USA) and miRNA transcriptome sequencing was performed by HiSeq 2500 sequencing system (Illumina) using the HiSeq Rapid Cluster Kit v2 (Illumina). Briefly, small RNA molecules from five healthy controls and five LUAD patients were amplified in 17 cycles with the adaptor primers after the small RNA (18–30 bp) was filtered by PAGE gel and ligated to 3′ adaptor system and 5′ adaptor mix system under optimal reaction conditions. Subsequently, the PCR products were purified with PAGE gel, and the average molecule length was determined using the Agilent 2100 bioanalyzer instrument (Agilent DNA 1000 Reagents) and quantified by real‐time qPCR (hydrolysis probes). The eligible libraries were amplified on cBot to produce the cluster on the flow cell, which was sequenced (single‐end) with the HiSeq 2500 System, following the manufacturer's illustration. The 50‐nt sequence tags from the deep sequencing were filtered by removing the 5′ and 3′ adapter sequences, low‐quality reads, and redundancy contaminants to obtain the clean reads. After quantity control program, the clean reads were mapped to Homo sapiens using Burrows‐Wheeler Alignment Tool, and their expression and distribution patterns were analyzed using the Short Oligonucleotide Alignment Program 19. Then, the clean reads were classified and annotated by screening against miRBase version 22.0 database 5. The quantity of known miRNAs is normalized and showed as reads per million (RPM) using the following computational formula: RPM = (number of reads mapping to miRNA/number of reads in clean data)×10^6^. EdgeR package 20 was used to analyze the significant difference between healthy voltmeters and LUAD patients and calculate *P*, using │log_2_ (fold change)│> 1 and *P* < 0.05 as the default threshold for significantly differential expression. All the data were presented in the GEO database (Accession number: GSE111803).

### Quantitative real‐time PCR

A hydrolysis probe–based RT‐qPCR assay for EV miRNAs was performed following the manufacturer's protocol (Applied Biosystems, TaqMan miRNA Assays, Shanghai, China). Briefly, 2 µL of extracted RNA from each sample, 3.5 μL of diethyl pyrocarbonate‐treated water, 2 μL of 5× reverse transcription buffer, 1 μL of 10 mm deoxyribonucleotide triphosphates (dNTPs), 0.5 μL of avian myeloblastosis virus reverse transcriptase (TaKaRa, Tokyo, Japan), and 1 μL of a stem‐loop RT primer (Applied Biosystems) were mixed and incubated at 16 °C for 30 min, 42 °C for 30 min, and 85 °C for 5 min. For real‐time PCR, a total of 20 μL of reaction mixture containing 1 μL of cDNA, 0.3 μL of Taq polymerase, 0.33 μL of hydrolysis probe (Applied Biosystems), 1.2 μL of 25 mm MgCl_2_, 0.4 μL of 10 mm dNTPs, 2 μL of 10× PCR buffer, and 14.77 μL of Milli‐Q water was incubated at 95 °C for 5 min, followed by 40 cycles at 95 °C for 15 s, 60 °C for 30 s, and 72 °C for 30 s using an ABI7300 (Applied Biosystems). All reactions were analyzed in triplicate, and the data were analyzed using the comparative Cq method, with cel‐miR‐39 as the exogenous control 13. Receiver operating characteristic (ROC) curve analysis was utilized to estimate the diagnostic value of plasma EV miRNAs according to the previous report 21.

### Expression and statistical analyses

spss 18.0 software (IBM, Almonk, NY, USA) was used for statistical analyses, and graphpad prism 6.0 (GraphPad Software, San Diego, CA, USA) was used to generate the graphs. The Mann–Whitney *U* test was used to compare significant differences in plasma exosomal miRNA expression between the LUAD patients and healthy volunteers. Spearman correlation analysis was performed to confirm the correlations between miRNAs and clinical indicators. Additionally, logistic regression was used to develop a combined miRNA panel to predict the probability of LUAD according to the method previously described 22, 23. A *P*‐value of < 0.05 was regarded as statistically significant.

## Results

### Characterization of plasma extracellular vesicles

**Figure 1:**
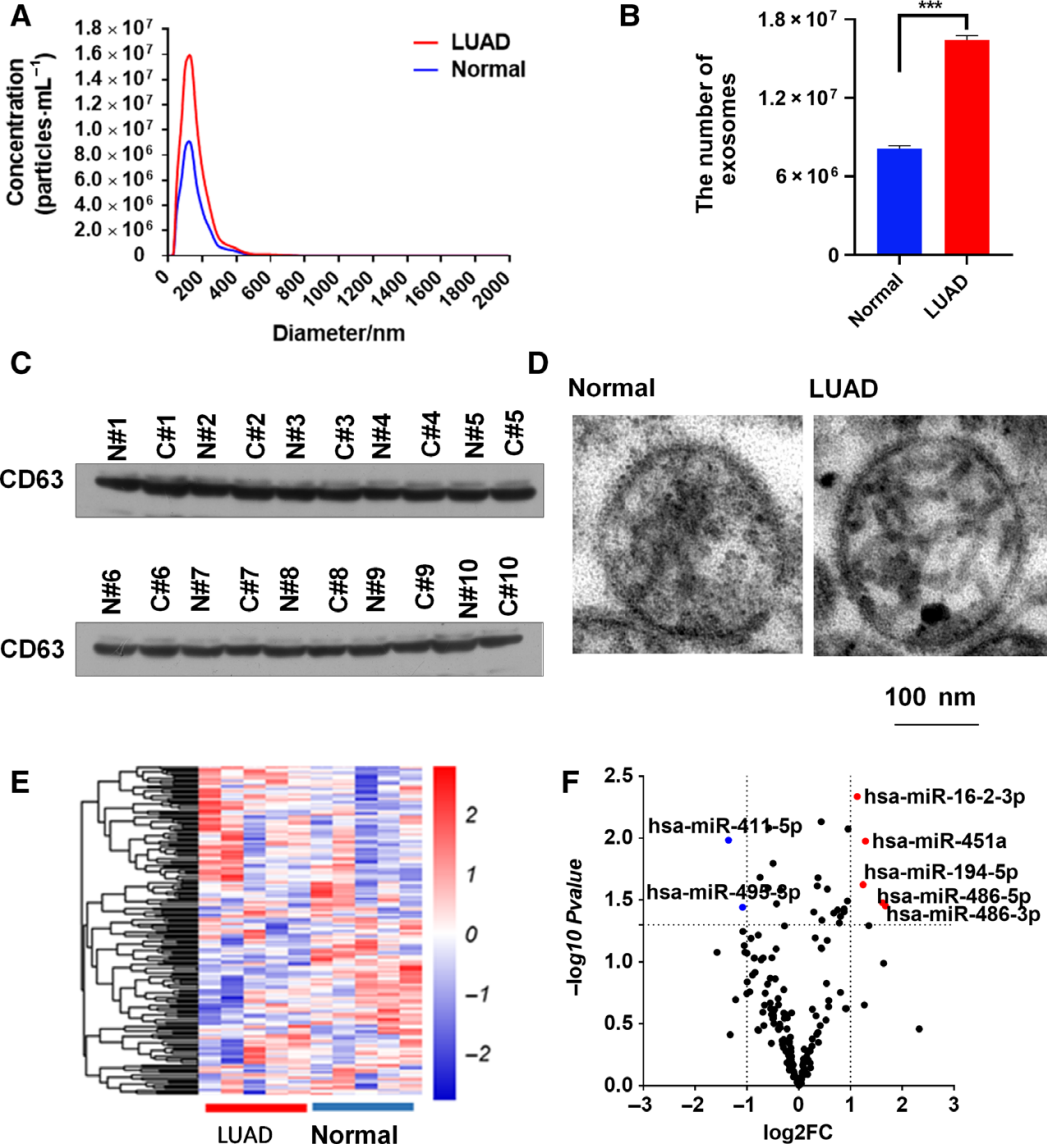
Identification of miRNAs in plasma EVs from LUAD patients and healthy volunteers in the screening phase. (A, B) The size of EVs derived from 10 LUAD patients and 10 healthy volunteers was analyzed by nanoparticle tracking technology. A: Representative image; B: Quantitative analysis. Data are presented as the mean ± s.e.m. and represent the mean concentration of EVs from 10 LUAD patients and 10 healthy volunteers. ***P < 0.001 as determined by two‐tailed *t*‐test. (C) Western blots of CD63 in EVs. (D) The shape and structure of EVs under TEM. The scale bars represent 100 nm. (E) Heatmap of miRNAs in plasma EVs from five normal controls and five LUAD patients. Red indicates higher expression in LUAD, and blue indicates lower expression in LUAD. (F) Volcano plots of miRNAs in plasma EVs from five normal controls and five LUAD patients. Red indicates upregulated miRNAs in LUAD, and blue indicates downregulated miRNAs in LUAD.

To analyze the efficacy of the plasma EVs, NTA, TEM, and western blotting analysis were used to characterize the EVs isolated from 20 randomly chose plasma samples (including 10 healthy volunteers and 10 LUAD patients). The diameter of the EVs was approximately 100 nm, as determined by NTA (Fig. 1A,B). As previously reported, the concentration of EVs in the plasma of LUAD patients was slightly higher than that in the plasma of healthy volunteers 9 (Fig. 1A,B). Through western immunoblotting analysis (Fig. 1C), the isolated EVs from the plasma of LUAD patients were further characterized by evaluating the expression of CD63 (an established marker for exosomes). The EVs were visualized by TEM and the typical size and rounded membrane‐bound morphology of EVs were observed (Figs 1D, S1 and S2). These results demonstrated that we successfully extracted the EVs from plasma.

### Plasma EV miRNA profiles analyzed by Illumina HiSeq 2500

In order to identify the appropriate miRNAs in the EVs used for LUAD diagnosis, a multiphase case–control study was designed to identify differentially expressed exosomal miRNAs in LUAD patients and healthy volunteers, as shown in the overview of the protocol in Fig. S3. The miRNAs in EVs from the plasma of five LUAD patients and five healthy volunteers wer profiled by Illumina HiSeq 2500 analysis (GSE111803). The heatmap and volcano plot revealed distinct expression patterns of plasma exosomal miRNAs between LUAD patients and healthy volunteers (Fig. 1E,F). A miRNA was considered to be a potential biomarker if the mean number of reads was > 100 and the fold change was ≥ 2‐fold between LUAD patients and healthy volunteers (*P* < 0.05). Consequently, five upregulated miRNAs (miR‐16‐3p, miR‐451a, miR‐194‐5p, miR‐486‐5p, and miR‐486‐3p) and two downregulated miRNAs (miR‐411‐5p, miR‐495‐3p) were identified as statistically significant (*P* < 0.05, as list in Table 2).

**Table 2:** Selection criteria of seven miRNAs from the screening phase.

miRNA_ID	Fold change	*P*
miR‐16‐3p	2.19	0.00
miR‐451a	2.45	0.01
miR‐194‐5p	2.37	0.02
miR‐486‐5p	3.08	0.03
miR‐486‐3p	3.19	0.04
miR‐411‐5p	0.39	0.01
miR‐495‐3p	0.47	0.04

### Plasma EV miRNA profiling during the training set

**Figure 2:**
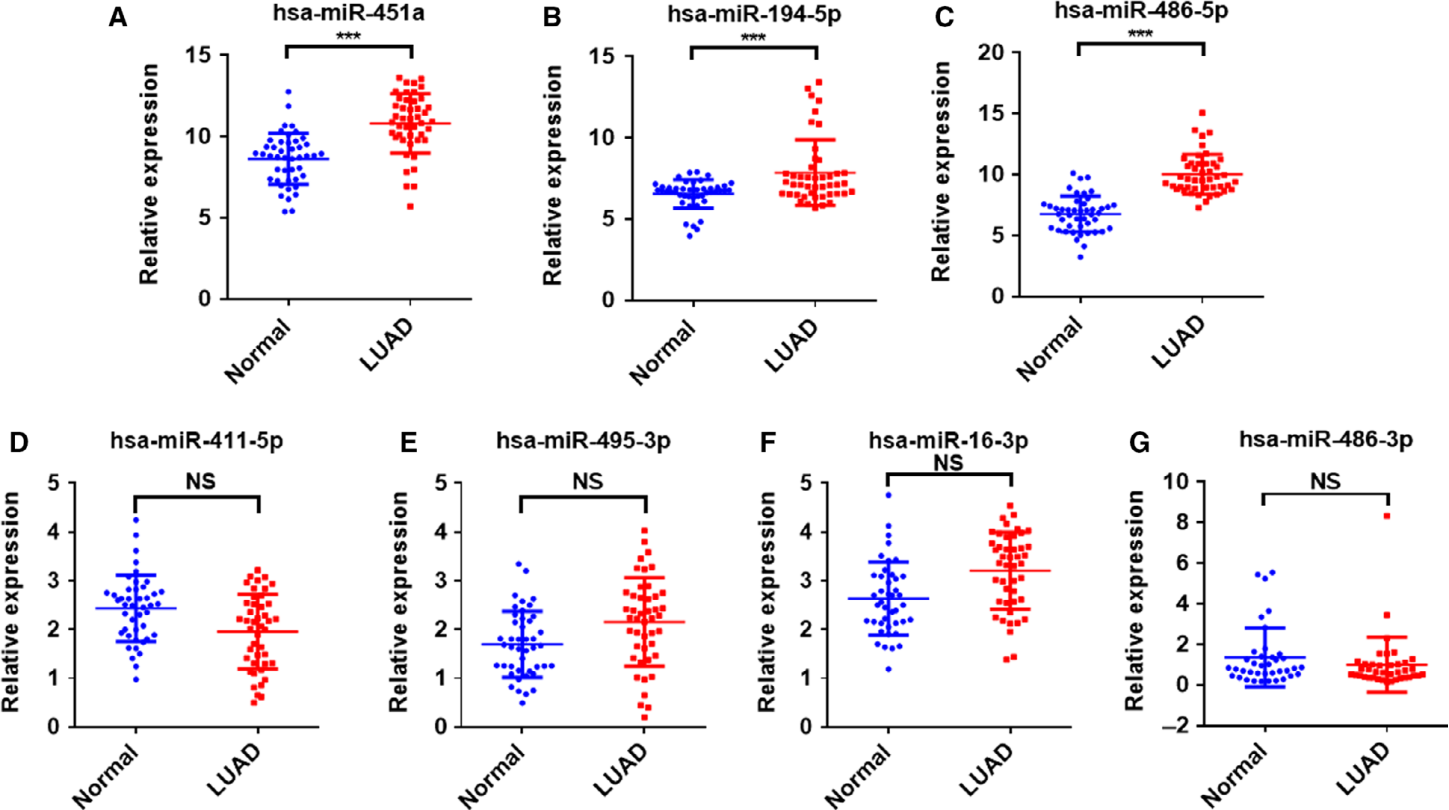
Plasma EV miRNA expression signature for LUAD diagnosis in the training phase. (A‐G) The expression of miR‐451a, miR‐194‐5p, miR‐486‐5p, miR‐411‐5p, miR‐495‐3p, miR‐16‐3p, and miR‐486‐3p in plasma EVs from patients with LUAD (*N* = 45) and normal samples from healthy volunteers (*N* = 45) by qRT‐PCR. Each point represents the mean of triplicate samples. Each *P*‐value was calculated with a nonparametric Mann–Whitney test. ***P < 0.001. NS: no significant difference.

The above observations by the Illumina HiSeq 2500 were further validated by qRT‐PCR to analyze the expression patterns of the potential miRNA candidates from the plasma of LUAD patients (*n* = 45) and healthy volunteers (*n* = 45). To determine the specificity and the concentrations of miR‐451a, miR‐194‐5p, and miR‐486‐5p, no‐template controls and synthetic single‐stranded miRNA were serially diluted and assessed using the qRT‐PCR assay to generate a standard curve. All the three miRNAs were consistently and efficiently amplified (Fig. S4A–C). We found that the levels of miR‐451a, miR‐194‐5p, and miR‐486‐5p in EVs were significantly increased in LUAD patients over healthy controls (*P* < 0.001, Fig. 2A–C). However, the other four miRNAs (miR‐16‐3p, miR‐411‐5p, miR‐495‐3p, and miR‐486‐3p) showed no difference between LUAD patients and healthy volunteers (*P* > 0.05, Fig. 2D–G). These results suggest that miR‐451a, miR‐194‐5p, and miR‐486‐5p may serve as useful and stable diagnostic biomarkers for LUAD.

### Validation and blinded testing of the EV miRNA signature for LUAD

**Figure 3:**
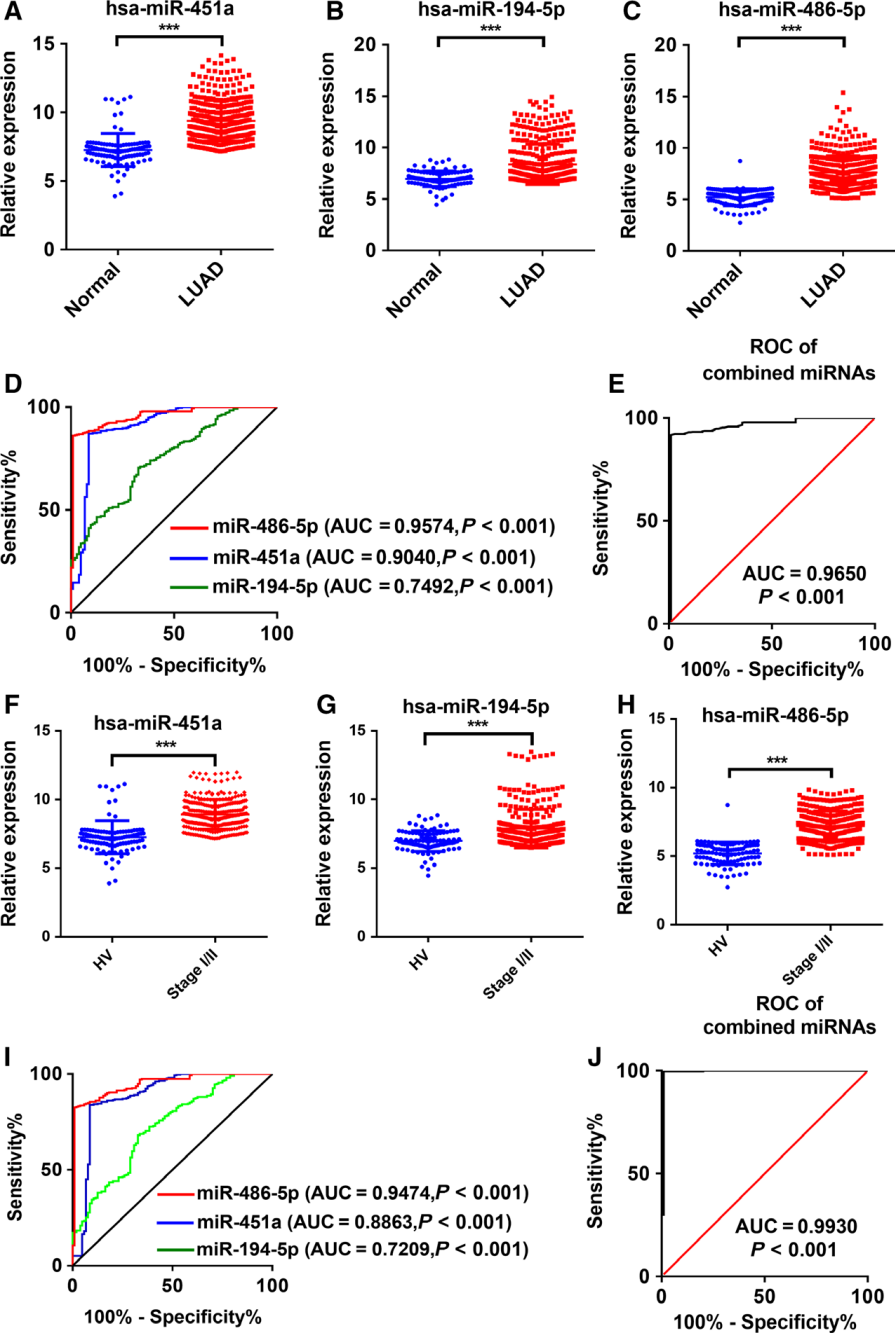
Plasma EV miRNA expression signature for LUAD diagnosis in the validation phase. (A–C) The expression of miR‐451a, miR‐194‐5p, and miR‐486‐5p in the plasma EVs from patients with LUAD (*N* = 389) and normal samples from healthy volunteers (*N* = 104) by qRT‐PCR. (D) ROC curve analysis for miR‐451a, miR‐194‐5p, and miR‐486‐5p in plasma EVs from patients with LUAD (*N* = 389) and normal samples from healthy volunteers (*N* = 104). (E) ROC curve analysis for the combined miRNA panel in plasma EVs from patients with LUAD (*N* = 389) and normal samples from healthy volunteers (*N* = 104). (F–H) The expression of miR‐451a, miR‐194‐5p, and miR‐486‐5p in plasma EVs from patients with stage I/II LUAD (*N* = 310) (stage I/II) and normal samples from healthy volunteers (*N* = 104) (HV) by qRT‐PCR. (I) ROC curve analysis for miR‐451a, miR‐194‐5p, and miR‐486‐5p in plasma EVs from patients with stage I/II LUAD (*N* = 310) and normal samples from healthy volunteers (*N* = 104). (J) ROC curve analysis for the combined miRNA panel in plasma EVs from patients with stage I/II LUAD (*N* = 310) and normal samples from healthy volunteers (*N* = 104). Each value is the mean ± SD; ***P < 0.001.

Next, we extended our studies in a larger cohort, comprising 389 LUAD patients and 104 healthy volunteers (Table 1), to validate the expression of miR‐451a, miR‐194‐5p, and miR‐486‐5p. Indeed, the concentrations of these 3 miRNAs in LUAD patients were significantly elevated over the levels detected in plasma‐derived from healthy volunteers in accordance with the results of the training set (*P* < 0.001, Fig. 3A–C). To investigate the diagnostic value of these three plasma EV miRNAs for LUAD, the ROC curve analysis was subsequently performed. As showed in Fig. 3D, the area under the curve (AUC) values of these miRNAs were 0.9040 (95% confidence interval [CI], 0.8633–0.9447) for miR‐451a, 0.7492 (95% CI, 0.6992–0.7992) for miR‐194‐5p, and 0.9574 (95% CI, 0.9378–0.9769) for miR‐486‐5p. On further analysis, we evaluated the diagnostic value of the combination of these miRNAs by a logistic regression model and found that the AUC was 0.9650 for the combination of these three miRNAs (*P* < 0.001, Fig. 3E). More interestingly, we also found the expression level of miR‐451a, miR‐194‐5p, and miR‐486‐5p in the EVs of plasma were gradually increased with the development of LUAD (Fig. S5)**.** Since the survival of LUAD patients is closely associated with the tumor stage at initial diagnosis, the diagnostic value of these three plasma EV miRNAs for stage I/II LUAD was analyzed. As showed in Fig. 3F–H, the concentrations of these three miRNAs were also significantly upregulated in the EVs derived from plasmas of stage I/II LUAD patients. The AUC values of these miRNAs for stage I/II LUAD were 0.8863 (95% CI, 0.8399–0.9327) for miR‐451a, 0.7209 (95% CI, 0.6643–0.7775) for miR‐194‐5p, and 0.9474 (95% CI, 0.9241–0.9706) for miR‐486‐5p (Fig. 3I). The AUC of the combination of these miRNAs was 0.9930 (Fig. 3J). These results reconfirmed that plasma EV miR‐451a, miR‐194‐5p, and miR‐486‐5p have relatively high diagnostic accuracy for LUAD, especially for the early diagnosis. The predictive value of the miRNA signature was further assessed in a blinded study, including 39 LUAD patients and 39 healthy volunteers. Based on the previous data, a signature composed of these three miRNAs (miR‐451a, miR‐194‐5p, and miR‐486‐5p) correctly discriminated 37 of the 39 LUAD samples (94.9% sensitivity) and 38 of the 39 normal samples. In summary, our data serve as proof of concept that the panel containing these three EV miRNAs may be a potential noninvasive biomarker for LUAD.

### Comparison of EV miRNAs before and after surgery

**Figure 4:**
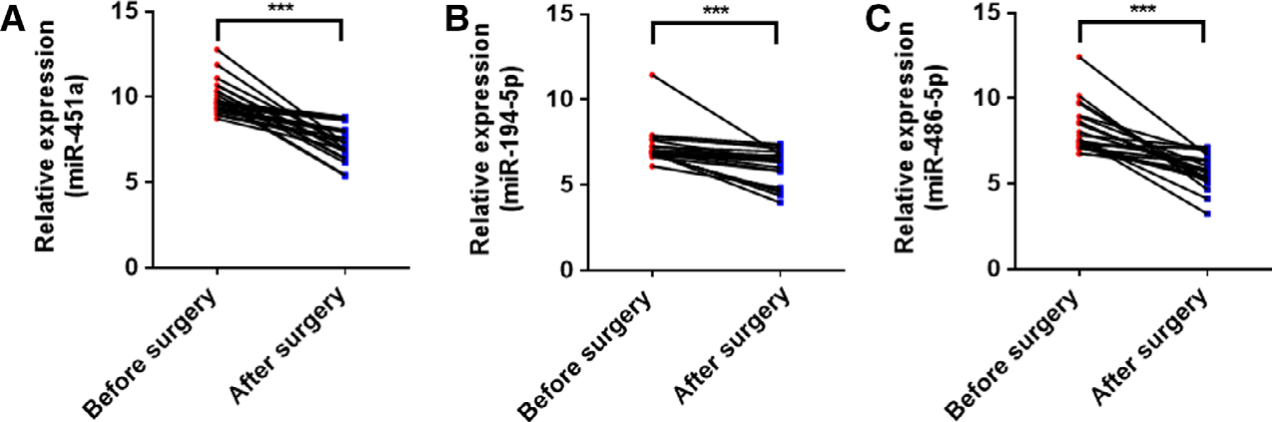
Plasma EV miRNA expression signature of LUAD patients before surgery and after surgery. (A–C) The expression of miR‐451a, miR‐194‐5p, and miR‐486‐5p in plasma EVs from 23 stage I LUAD patients before surgery and 1 month after surgery by qRT‐PCR. Each value is the mean ± SD; ***P < 0.001.

We also did some preliminary analysis to investigate whether these plasma EV miRNAs are valuable for treatment monitoring in LUAD patients. We collected paired plasma samples from 23 stage I LUAD patients before and after surgery. It is interesting to note that the levels of these three EV miRNAs were decreased 1 month after surgery (Fig. 4A‐C), highlighting the clinical potential for monitoring LUAD.

## Discussion

The survival of LUAD patients is closely related to the tumor stage at initial diagnosis. Recent approaches for the diagnosis of LUAD involve noninvasive methods and invasive methods, which expose patients to ionizing radiation or have low sensitivity 4. Earlier studies have suggested that EVs could serve as a delivery system in cells, tissues, and organs, and regulate a widespread biological process through intercellular communication 24. Furthermore, an increasing number of studies have revealed that EVs secreted by cancer cells can be released and transport tumor cell miRNAs into the circulation, and could be anticipated to be potentially useful biomarkers for tumors 8.

In order to explore whether EV miRNAs in the plasma could be clinically applicable diagnostic biomarkers for LUAD early diagnosis, we designed a multiphase case–control study. In this study, we developed a reliable strategy that combines techniques of high‐throughput sequencing to screen candidate miRNAs and RT‐qPCR assays to validate candidate miRNAs in 496 LUAD patients (395 stage I/II patients and 101 stage III/IV patients). The levels of EV miR‐451a, miR‐194‐5p, and miR‐486‐5p in the plasma were found to be significantly increased in patients with LUAD, compared to the healthy volunteers. In agreement with our observations, several studies have reported that miR‐451a, miR‐194‐5p, and miR‐486‐5p were dysregulated in plasma/serum from lung cancer patients 25, 26, 27, 28, 29, 30. However, the samples used in most of these studies are late‐stage LUAD patients. In our study, 80% of the recruited patients were in early stage. More interestingly, we found these miRNAs levels were gradually increased with the development of LUAD. Additionally, the levels of these three miRNAs were markedly decreased 1 month after surgery.

Most cell types are capable of forming and secreting EVs as a method of intercellular communication. EVs have been shown to contain proteins and RNAs, especially miRNAs, that can be transferred to other cells and thereby trigger a broad range of cancer‐related cellular events, including cell growth, invasion, metastasis, and immune escape 9, 24. Therefore, it is natural to speculate that miR‐451a, miR‐194‐5p, and miR‐486‐5p, which are significantly increased in the EVs of plasma from LUAD patients, may also be involved in the occurrence and development of LUAD. However, as previously reported 31, 32, 33, 34, 35, the expression of miR‐451a, miR‐194‐5p, and miR‐486‐5p was significantly downregulated in LUAD tissues compared to that in normal tissue in the TCGA database (Fig. S6). Several studies have confirmed that miR‐451a, miR‐194‐5p, and miR‐486‐5p might inhibit tumor cell growth by suppressing proliferation and invasion or promote apoptosis by targeting cancer‐associated genes 31, 32, 33, 34, 35. Recently, some studies found that tumor suppressor gene‐induced senescent cells modulate the immune response, which promotes the establishment of the inflammatory microenvironment that contributes to metastasis 36. The abovementioned miRNAs might be secreted from tumor tissue and be involved in the immune escape of cancer cells, which contributes to tumor metastasis. For example, miR‐194, a typical p53‐responsive miRNA, has been shown to trigger replicative senescence of mouse embryonic fibroblast cells by potentially inhibiting DNMT3A expression 37. Therefore, tumor suppressor miRNAs released from LUAD may also play a major role in inducing the senescence of cells in areas of proximal and distant tissues to accelerate the onset of the metastatic process of LUAD, while additional validation studies will be necessary to gain a better understanding of the potential biological events mediated by these miRNAs.

Our study also has some limitations. First, it could not assure that the pellets isolated from the plasma samples by the Total Exosome Isolation Kit were indeed exosomes, although we performed NTA, TEM, and western blotting analysis to characterize the isolated pellets from the plasma (the pellets were named as EVs, which included exosomes). Under this condition, the increase of miR‐451a, miR‐194‐5p, and miR‐486‐5p in the plasma EVs might not come from the LUAD tumor tissues. For example, miR‐451a has been thought to be upregulated in the blood under hemolysis, or came from the EVs of platelets 38, 39. In our study, the samples from LUAD patients and healthy volunteers are strictly transported under 4° C, and the plasma was isolated once the blood was collected to avoid the influence of hemolysis and activated blood cells/platelets on the exosomal miRNAs 40. Secondly, no benign or symptom similar patients are included in our study. Recently, Wang *et al*.^25^ found miR‐483‐5p in the exosomes derived from pleural effusions was preferentially represented in LUAD when compared to tuberculous and other benign lesions. These results indicate these three miRNAs in the EVs from plasma might likely distinguish LUAD patients from benign/ symptom similar patients. However, further study in a larger cohort is needed to confirm these findings.

Taken together, our findings indicate that a miRNA panel comprising miR‐451a, miR‐194‐5p, and miR‐486‐5p in plasma EVs has reasonably high sensitivity and specificity for distinguishing patients with LUAD from healthy controls, especially stage I/II LUAD patients. This panel has great potential as a noninvasive biomarker for early detection, as well as for routine screening of LUAD.

## Conflict of interest

The authors declare no conflict of interest.

## Author contributions

Quan Zhao designed the experiments. Bing Yao, Shuang Qu, Ruifeng Hu, Wen Gao, and Shidai Jin performed the experiments and analyzed the results. Ming Liu wrote the manuscript.

## Supporting information


**Fig. S1** **.** The shape and structure of EVs under TEM.
**Fig. S** **2** **.** The wide‐field TEM images of EVs derived from plasma of healthy volunteers (A: Normal) and patients with LUAD (B: Cancer).
**Fig. S3** **.** An overview of the experimental design.
**Fig. S4** **.** Standard curves of miR‐451a, miR‐194‐5p, and miR‐486‐5p using synthetic miRNAs.
**Fig. S5** **. **The expression level of miR‐451a, miR‐194‐5p, and miR‐486‐5p in the EVs of plasma from healthy volunteers (HV) and LUAD patients at different stages (I: stage I; II: stage II; III: stage III; IV: stage IV).
**Fig. S6** **.** The expression levels of miR‐451a, miR‐194‐5p, and miR‐486‐5p in the LUAD tissues (C) and normal tissues (N) in the TCGA database. ***P < 0.001.

## Data Availability

All data generated or analyzed during this study are included in this published article. Raw and processed data are stored in the laboratory of GB and are available upon request. This RNA‐sequencing data are deposited in the NCBI Gene Expression Omnibus (GEO) datasets under the accession number GSE111803 (www.ncbi.nlm.nih.gov/geo).
