# Supplementary material for: A panel of miRNAs derived from plasma extracellular vesicles as novel diagnostic biomarkers of lung adenocarcinoma
Source: FEBS Open Bio. 2019 Nov 21;9(12):2149–58. doi: 10.1002/2211-5463.12753 (PMC6886307; doi:10.1002/2211-5463.12753)
Supplement: Supplementary file 1 — Fig. S1 . The shape and structure of EVs under TEM. Fig. S 2 . The wide‐field TEM images of EVs derived from plasma of healthy volunteers (A: Normal) and patients with LUAD (B: Cancer). Fig. S3 . An overview of the experimental design. Fig. S4 . Standard curves of miR‐451a, miR‐194‐5p, and miR‐486‐5p using synthetic miRNAs. Fig. S5 . The expression level of miR‐451a, miR‐194‐5p, and miR‐486‐5p in the EVs of plasma from healthy volunteers (HV) and LUAD patients at different stages (I: stage I; II: stage II; III: stage III; IV: stage IV). Fig. S6 . The expression levels of miR‐451a, miR‐194‐5p, and miR‐486‐5p in the LUAD tissues (C) and normal tissues (N) in the TCGA database. ***P < 0.001. [file FEB4-9-2149-s001.doc]

**
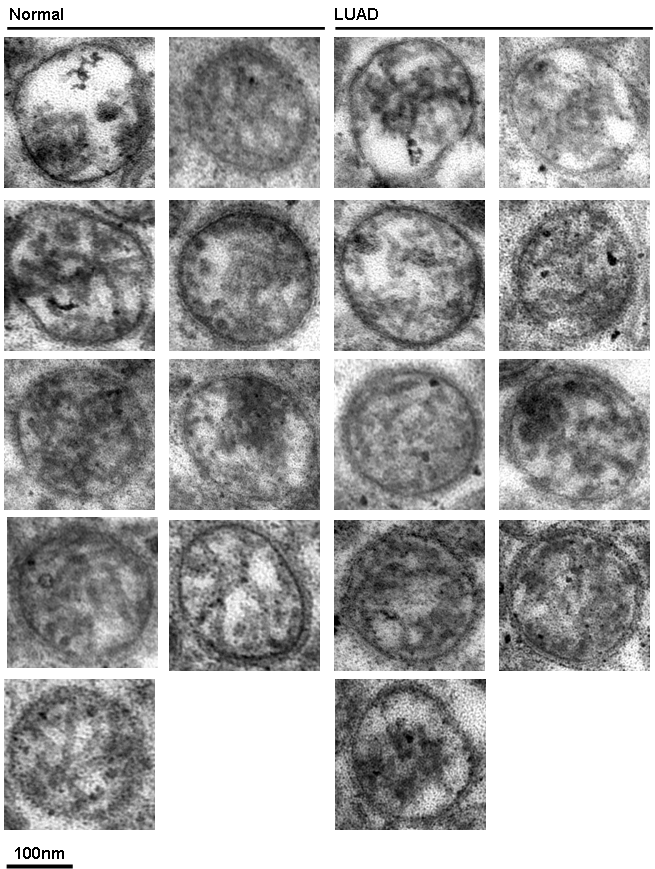
**

**Figure s1. The shape and structure of EVs under TEM.**

**
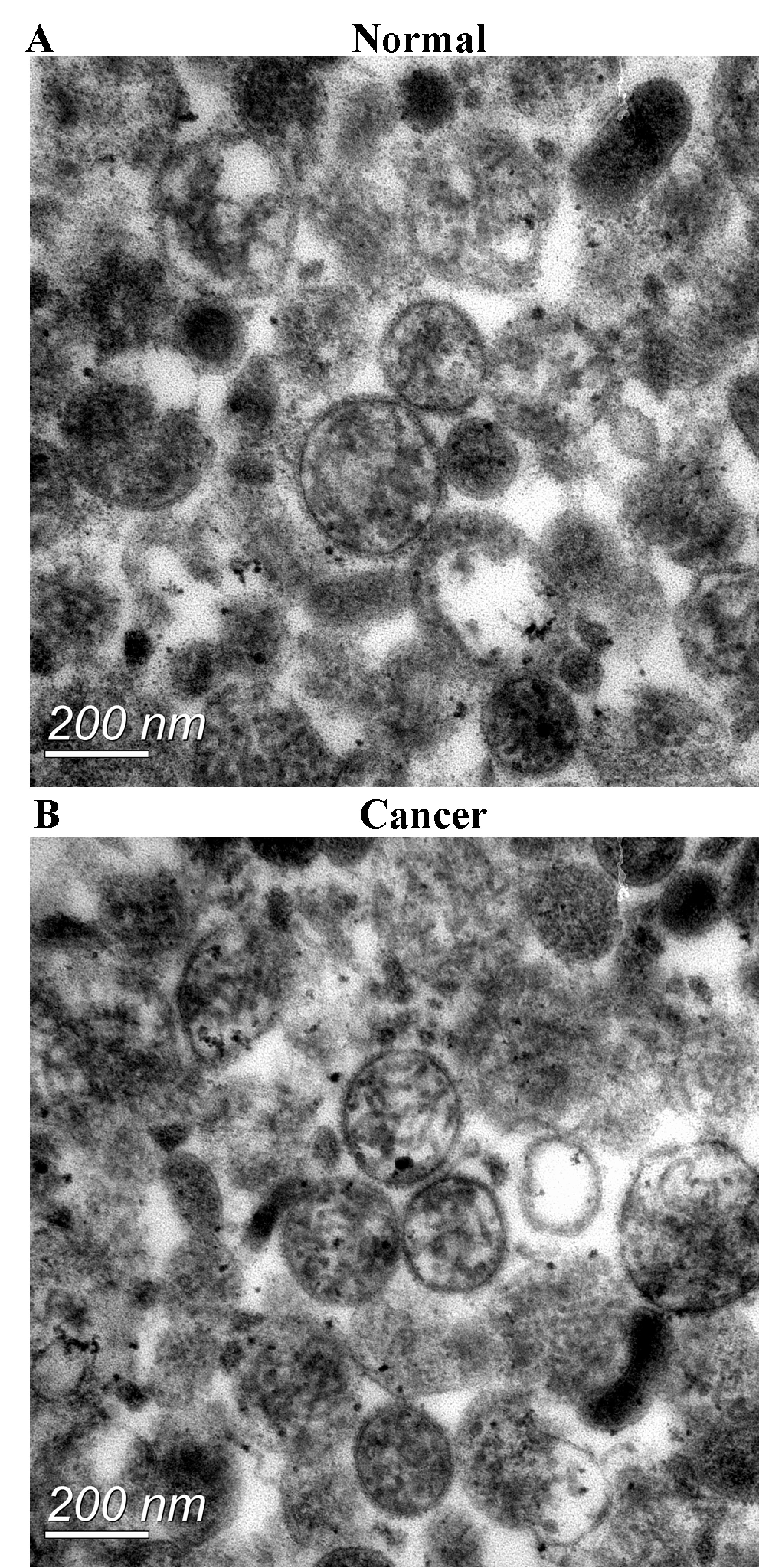
**

**Figure s2. The wide field TEM images of EVs derived from plasma of healthy volunteers (A: Normal) and patients with LUAD (B: Cancer).**

**
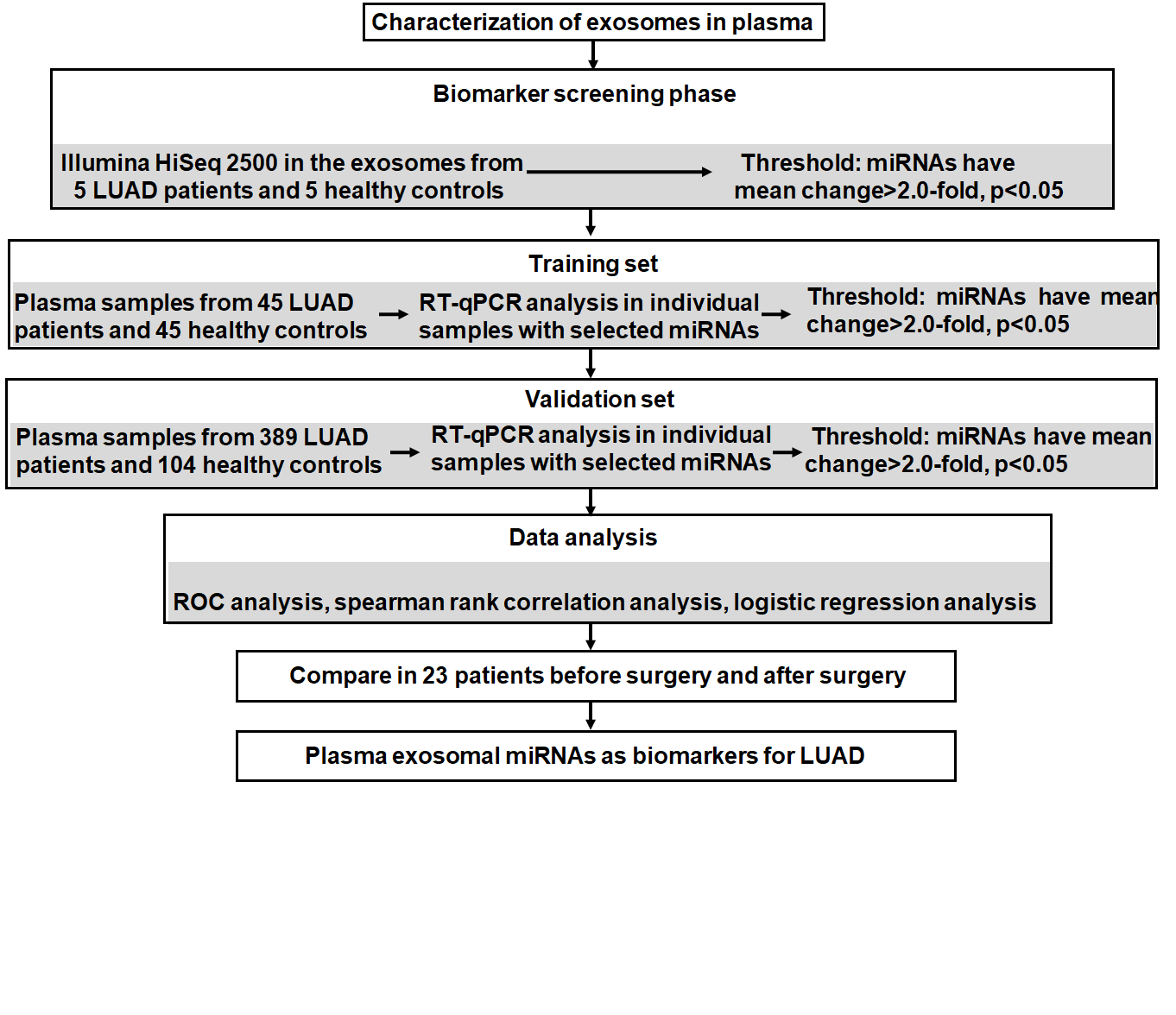
**

**Figure s3. An overview of the experimental design.**

**
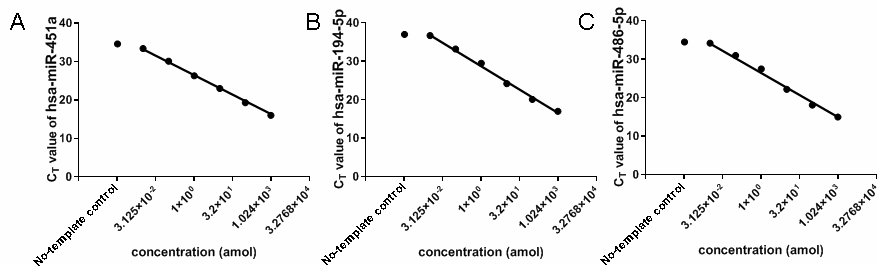
**

**Figure s4. Standard curves of miR-451a, miR-194-5p, and miR-486-5p using synthetic miRNAs.**

**
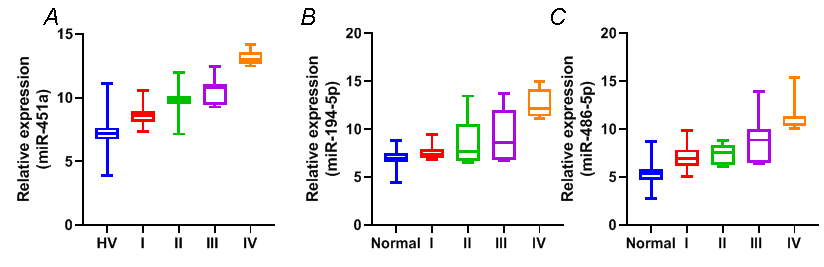
**

**Figure s5. The expression level of miR-451a, miR-194-5p, and miR-486-5p in the EVs of plasma from healthy volunteers (HV) and LUAD patients with different stage (I: stage I; II: stage II; III: stage III; IV: stage IV).**

**
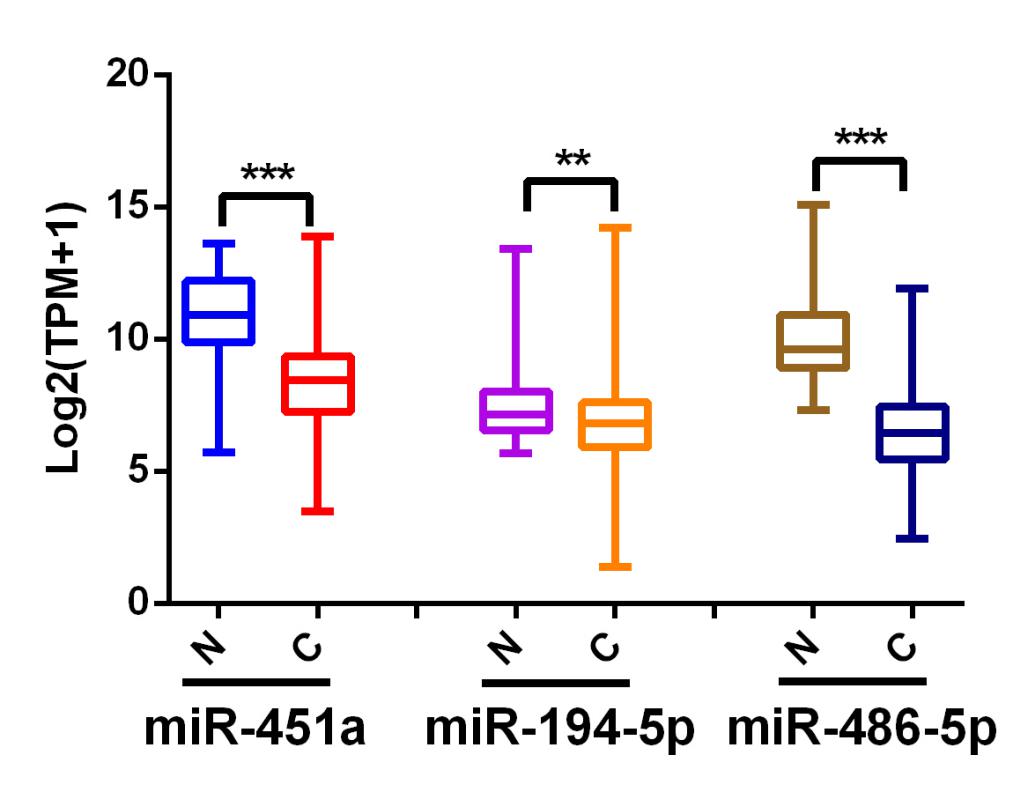
**

**Figure s6. The expression level of** **miR-451a, miR-194-5p, and miR-486-5p in the LUAD tissues (C) and normal tissues (N) in the TCGA database. ***P <0.001.**
